# Thrombotic Versus Bleeding Risk After Transcatheter Aortic Valve Implantation

**DOI:** 10.3390/jcm15051767

**Published:** 2026-02-26

**Authors:** Kotsi Sofia Fotoula, Abdalazeem Ibrahem, Allam Harfoush, Hussain Hussain, Ammar Ezeldin, Hilal Khan, Diana A. Gorog, Mohamed Farag

**Affiliations:** 1 Cardiology Department, North Bristol NHS Trust, Bristol BS10 5NB, UK; fotoula.kotsi@nbt.nhs.uk (K.S.F.); abdalazeem.ibrahem2@nhs.net (A.I.); allam.harfoush@nbt.nhs.uk (A.H.); 2 School of Life and Medical Sciences, University of Hertfordshire, Hatfield AL10 9AB, UK; d.gorog@imperial.ac.uk; 3 The Faculty of Health, Medicine, and Society, University of Chester, Chester CH1 4BJ, UK; 4 Cardiology Department, Grange University Hospital, Cwmbran NP44 8YN, UK; drhussain_h@yahoo.com; 5 Queens Elizabeth Hospital, Gateshead, Newcastle Upon Tyne NE9 6SX, UK; ammarezeldin2017@gmail.com; 6 Cardiothoracic Department, Freeman Hospital, Newcastle Upon Tyne NE7 7DN, UK

**Keywords:** transcatheter aortic valve implantation (TAVI), aortic stenosis, antithrombotic therapy, bleeding risk, thrombotic complications, leaflet thrombosis

## Abstract

**Background:** Transcatheter aortic valve implantation (TAVI) is increasingly used across all risk groups, meaning more patients are living long-term with transcatheter bioprosthetic valves. These patients are often multimorbid and vulnerable to both thrombotic and bleeding complications. Optimal antithrombotic therapy remains uncertain due to differences in trial design, patient demographics, and procedural practices. **Methods:** We undertook a narrative review that included randomised controlled trials, observational studies, biomarker research, and guideline recommendations on post-TAVI antithrombotic therapy. We evaluated the available evidence for antiplatelet and anticoagulant strategies after TAVI, predictors of bleeding and thrombotic complications, to identify emerging approaches using biomarkers for personalised risk stratification. **Results:** Thrombotic events after TAVI are predominantly early and procedural in origin, while new-onset atrial fibrillation (AF) leads to substantial late risk. Subclinical leaflet thrombosis is frequent, but its clinical significance remains uncertain, as anticoagulation reduces CT-detected leaflet abnormalities without improving clinical outcomes. Early bleeding within the first 30 days remains a principal contributor to mortality, influenced by frailty, vascular access, comorbidity, and intensity of antithrombotic therapy. Randomised evidence consistently supports a minimalist, indication-driven regimen: single antiplatelet therapy for patients without an oral-anticoagulation (OAC) indication, and OAC monotherapy for those with AF. Routine OAC use in unselected patients carries no advantage and exposes them to harm. Biomarkers and machine-learning models show promise for future individualised risk assessment. **Conclusions:** Antithrombotic strategies post-TAVI should prioritise minimising bleeding while maintaining adequate thromboembolic protection. Single antiplatelet therapy for patients without an indication for OAC and OAC alone for those with AF offer the best balance of safety and efficacy. Ongoing trials may clarify the role of imaging-guided therapy and biomarker-based risk stratification and refine antithrombotic strategies.

## 1. Introduction

Aortic stenosis (AS) is one of the most common valvular heart conditions in developed countries [1]. The degenerative–calcific process leads to progressive valve narrowing and left ventricular pressure overload and, once symptoms develop, is associated with high mortality, if left untreated [2]. Transcatheter aortic valve implantation (TAVI) has transformed the management of severe AS, offering a less invasive alternative to surgery and expanding rapidly from inoperable and high-risk patients to those at intermediate and even lower surgical risk [3,4]. As a result, the number of patients living long term with a bioprosthetic transcatheter valve is steadily increasing.

**Figure 1:**
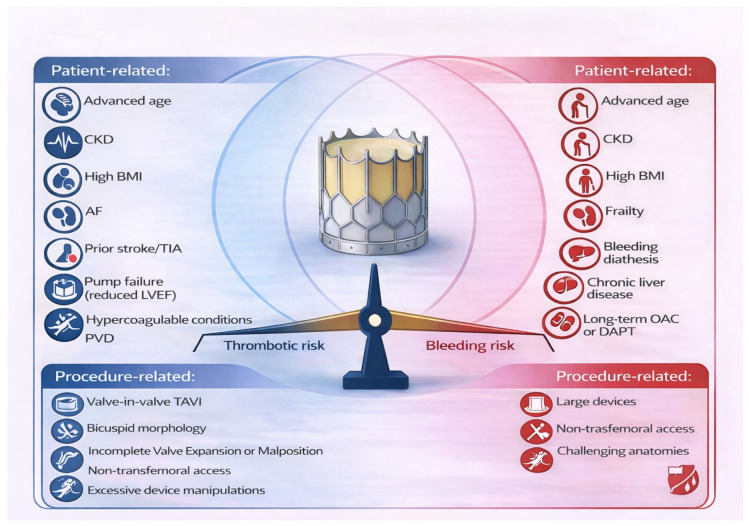
Central illustration: Bleeding and thrombotic risk in patients undergoing TAVI. Contributors to bleeding risk (Right) and thrombotic risk (Left) can overlap, highlighting the need for an individualised, balanced antithrombotic approach. AF: atrial fibrillation, BMI: body mass index, CKD: chronic kidney disease, DAPT: dual antiplatelet therapy, LVEF: left ventricular ejection fraction, OAC: oral anticoagulation, PVD: peripheral vascular disease, TIA: transient ischaemic attack.

TAVI recipients represent a population at particularly high risk for both thrombotic and bleeding complications. Advanced age, atrial fibrillation (AF), chronic kidney disease, and frailty contribute to a prothrombotic burden, while the procedure itself induces endothelial trauma, turbulent flow, and platelet activation [5]. Conversely, antithrombotic therapy and the high burden of comorbidities significantly increase the risk of major bleeding, including gastrointestinal and intracranial haemorrhage [6]. Many clinical and patient factors that increase the risk of thrombosis also increase the risk of bleeding, and this overlap makes managing antithrombotic therapy challenging, as the treatment for thrombosis inherently increases bleeding risk. This delicate balance makes selection and intensity of post-procedural antithrombotic therapy a central determinant of net clinical benefit (Figure 1).

Despite this, current antithrombotic strategies after TAVI remain largely empirical. Consensus documents and expert opinion generally recommend single antiplatelet therapy for patients in sinus rhythm and oral anticoagulation (OAC) alone for those with AF, or any other indication for anticoagulation [7]. Yet these broad categories inadequately reflect individual variation in thrombotic tendency (e.g., prior stroke, subclinical leaflet thrombosis, and complex aortic anatomy) and bleeding susceptibility (e.g., prior bleeding, anaemia, frailty, and concomitant need for dual antiplatelet therapy). Procedural variables, such as balloon aortic valvuloplasty, valve type and size, and access route, may further influence the risk of thromboembolism or valve thrombosis [8], but the evidence remains inconclusive. In parallel, imaging studies have highlighted phenomena such as hypoattenuated leaflet thickening (HALT) and subclinical leaflet thrombosis after TAVI, raising concerns about potential links to stroke, transient ischaemic attacks (TIAs), and accelerated valve degeneration [9]. These findings have further sparked interest in antithrombotic strategies to prevent leaflet pathology, but randomised trial results are inconclusive, with significant safety concerns for some agents and regimens. The conflict between imaging findings, clinical outcomes, and bleeding risk highlights the need for a more thorough framework.

A precision-medicine approach is therefore needed. Individualised risk-stratification tools that integrate clinical characteristics, procedural factors, imaging markers, and circulating biomarkers could enable tailored antithrombotic therapy, balancing both thrombotic and bleeding events. In this review, we summarise the evidence for antiplatelet and anticoagulant strategies after TAVI, critically appraise the strengths and limitations of current data, and discuss how emerging mechanistic insights might inform a more personalised, risk-adapted approach to antithrombotic management.

## 2. Methods

This narrative review was conducted through a structured literature search of PubMed/MEDLINE, ClinicalTrials.gov, the Cochrane Library, and Embase for studies published up to September 2025 investigating antithrombotic and anticoagulation regimens post-TAVI. Search terms included combinations of “TAV”, “TAVR”, “transcatheter aortic valve implantation”, “antithrobotic therapy”, “antiplatelet”, “oral anticoaguation”, “leaflet rhrombosis”, “bleeding”, “stroke”, and “biomarkers”. We included randomised controlled trials, large observational studies and meta-analyses relevant to thrombotic and bleeding risk post-TAVI. Emphasis was placed on recent trials informing current clinical practice. Guideline and consensus documents were identified from official publications of the European Society of Cardiology (ESC), American Heart Association (AHA), American College of Cardiology (ACC) and European Association for Cardiothoracic Surgery (EACTS). Study selection was based on clinical relevance, novel information presented, and strength and quality of evidence.

## 3. Thrombotic Risk After TAVI: Predictors and Mechanisms

Thrombotic complications following TAVI, including ischaemic stroke, valve thrombosis, and systemic embolism, remain major contributors to post-procedural morbidity and mortality. These arise from multifactorial causes involving patient-related, device-specific, and procedural factors, including the development of new-onset AF [10,11,12].

## 4. Ischaemic Stroke

Ischaemic stroke remains a serious complication following TAVI, with an incidence of approximately 1.5% within 24 h, increasing to 3–4% by 30 days, and reaching 5–8% by one year [10,11,12]. Approximately 34% of strokes occur on the day of the procedure, and 80% within the first week [10], confirming the predominance of early procedural mechanisms.

Most early strokes are thought to be caused by procedural emboli. This is driven by several intraoperative factors, including balloon pre- and/or post-dilatation, manipulation of stiff guidewires, and positioning of the new valve, which may embolise calcific material from the diseased aortic valve or arch [10]. Imaging studies and cerebral protection filter analyses have confirmed that these steps can release calcified and atheromatous debris, contributing directly to stroke pathogenesis. Independent predictors of early cerebrovascular events also include advanced age, prior stroke or TIA, chronic kidney disease (CKD, eGFR < 30 mL/min/1.73 m^2^), and AF [10].

Device selection and access route may influence stroke incidence. For instance, transapical implantation using the Edwards SAPIEN valve has been associated with the lowest 30-day stroke/TIA rate (~2.7%), whereas retrograde transfemoral implantation with the same valve produced higher rates (~4.2%). Interestingly, the use of sheath-protected delivery systems was associated with a lower stroke rate (~3.1%) than unshielded larger-profile devices [12].

## 5. Atrial Fibrillation

New-onset atrial fibrillation (NOAF) occurs in approximately 10% of patients undergoing TAVI and has been consistently associated with worse clinical outcomes [13]. NOAF after TAVI has been linked to a significantly increased risk of mortality, stroke, major bleeding, permanent pacemaker implantation (PPM), acute kidney injury (AKI), and prolonged hospital stay [13,14]. Risk factors for its development include a higher Society of Thoracic Surgeons (STS) score, CKD, peripheral vascular disease, severe mitral regurgitation, pulmonary hypertension, and transapical access [13].

In contrast, pre-existing AF is primarily associated with AKI and early bleeding, but not with stroke, late bleeding, or increased mortality [14]. This suggests that NOAF may carry a more detrimental short-term prognosis compared to pre-existing AF. It remains unclear whether AF directly contributes to these adverse events or reflects a background of underlying frailty, comorbidity and/or chronic OAC use. Alternatively, the development of NOAF may reflect a more challenging perioperative course, serving as a marker of patients already predisposed to poorer post-procedural outcomes [14]. It is worth noting that the CHA_2_DS_2_-VASc score used to assess stroke risk in patients with AF incorporates several variables, such as advanced age, diabetes, vascular disease, and heart failure, all of which are prevalent in the TAVI population. This overlap complicates attributing causality to AF alone.

Interestingly, the PARTNER 3 trial assessed outcomes in patients with severe AS (mean age: under 73 years, with low surgical risk) and found an 8% incidence of NOAF post-TAVI. Early NOAF, typically defined as AF occurring during hospitalisation or within 30 days post-procedure, was not predictive of stroke or mortality, and late NOAF (occurring after discharge) was associated with a nearly ninefold increased risk of poorer outcomes, including death, stroke, or rehospitalisation [15].

These findings support the hypothesis that procedure-related mechanisms, such as inflammation, oxidative stress, or sympathetic activation, contribute to early NOAF that might subside. In contrast, late NOAF may reflect a more sustained atrial remodelling process and thus portend a higher long-term risk. Importantly, decisions regarding long-term anticoagulation should be guided by validated stroke risk assessments, such as the CHA_2_DS_2_-VASc score, rather than solely on AF onset or imaging findings.

## 6. Silent Cerebral Ischaemia

Beyond clinical stroke, silent ischaemic brain lesions are highly prevalent following TAVI. Diffusion-weighted magnetic resonance imaging (MRI) has shown such lesions in 60–90% of patients, often without overt neurological symptoms [16,17]. These are believed to result from procedural embolization, particularly during arch navigation, wire placement, balloon dilatation, and valve deployment. While most lesions resolve on imaging within 3 months [12], there may be an association with cognitive impairment, prompting interest in cerebral protection devices, though conclusive benefits have not yet been established [17,18].

## 7. Leaflet Thrombosis

Subclinical leaflet thrombosis (SLT) can be detected on cardiac computed tomography (CT) as HALT, with or without reduced leaflet motion (RLM), and is a relatively common occurrence post-TAVI. Definitions across contemporary studies describe SLT as the presence of a thrombus without associated clinical symptoms or haemodynamic deterioration [19,20,21]. In contrast, clinical valve thrombosis (CVT) refers to cases with obstructive gradients, heart failure symptoms, or thromboembolic events.

The reported incidence of SLT ranges from 11% to 15%, while CVT remains rare, at around 1% [20,21]. Though initially considered benign, SLT has been associated with an increased risk of stroke in some analyses. One study showed that stroke risk was more than three times higher in patients with subclinical valve thrombosis (SCVT) compared to those without [21]. However, other pooled estimates suggest that this association may not always reach statistical significance when considering SLT alone [20]. In contrast, CVT is consistently associated with a significantly higher risk of cerebrovascular events [19,20,21].

SLT is commonly detected on routine CT imaging at 30 days post valve implantation. In the PARTNER 3 CT substudy, the incidence of HALT was 10% at 30 days, increasing to 24% at 1 year. It follows a dynamic pattern with half of the cases identified at 30 days resolving by 1 year, while new HALT develops in 21% of patients without initial imaging abnormalities. At an initial phase this was more frequent in TAVI patients but was not significant at 1 year [22].

Predictors of leaflet thrombosis include a larger prosthesis diameter, the use of balloon-expandable or valve-in-valve devices, single antiplatelet therapy, and an elevated BMI [19,20,23]. The type of pharmacotherapy influences risk: single- or dual-antiplatelet regimens were commonly used in patients who developed SLT, whereas OAC was protective across multiple studies, reducing both incidence and transvalvular gradients [21,23].

Choi et al. highlighted that thrombus formation is a common finding on imaging, with over 43% of patients demonstrating thrombosis at the aortic valve complex. A small maximum valve diameter and low body surface area were identified as predictors of complex or perivalvular thrombosis, while reduced renal function predicted leaflet thrombosis [19]. However, no association was found between thrombus presence (of any type or location) and the incidence of stroke, cerebral thromboembolism on MRI, neurologic dysfunction, or adverse clinical outcomes. This finding contrasts with earlier work suggesting that leaflet thrombosis was associated with an elevated stroke risk [23]. The ADAPT-TAVR trial supports these observations by demonstrating that while edoxaban significantly reduced HALT compared to DAPT, this reduction did not translate into a lower incidence of cerebral embolic events or neurologic outcomes [24].

Together, these studies suggest that although a subclinical thrombus is frequently visualised post-TAVI and may respond to anticoagulation, its clinical relevance remains uncertain. Some observational cohorts have reported an association between early HALT and increased long-term mortality (>2 years; however, causality has not been demonstrated). Notably, even though short-term warfarin treatment resulted in resolution of HALT, its presence at 30 days was still associated with increased mortality during follow-up [25]. Current evidence does not support routine CT screening or intensified antithrombotic therapy in the absence of clinical symptoms or haemodynamic compromise. Routine anticoagulation remains controversial but may be justified in selected high-risk populations.

## 8. Bleeding Risk After TAVI: Predictors and Mechanisms

Bleeding is a significant complication following TAVI, particularly in the early post-procedural period. Significant or life-threatening bleeding rates vary widely across trials, ranging from 2.4% to 41.7%, depending on patient population, procedural approach, and antithrombotic regimen [26]. In a large real-world cohort, major bleeding occurred in 6.6% of patients within 30 days, with rates falling from 11.5% in 2007–2010 to 5.5% in 2019–2022, likely reflecting improvements in operator experience, sheath size, transfemoral-first strategy, and the inclusion of relatively lower-risk patients [26,27].

Early bleeding (≤30 days) is most often access-site related and is typically driven by vascular trauma and procedural anticoagulation [27,28]. These events are associated with increased early mortality, prolonged hospitalisation, and the need for transfusion. Despite advances in procedural technique and valve technology, bleeding remains a key determinant of poor outcomes. Access-site bleeding alone accounts for up to 70% of major bleeds, while late events are more commonly gastrointestinal, urogenital, or intracranial in origin [27]. Routine or selective administration of protamine sulphate to reverse intraprocedural unfractionated heparin after valve deployment has been shown to reduce access-site bleeding events, potentially improving early bleeding outcomes. Non-access-site bleeding may carry greater prognostic weight, possibly reflecting underlying frailty or primary haemostatic disorders [29].

Frailty is a well-established independent predictor of major bleeding after TAVI [26,30]. This risk appears to be mediated through multiple mechanisms. Sarcopenia (e.g., reduced chair-rise performance) alters pharmacokinetics, leading to supratherapeutic plasma concentrations of hydrophilic anticoagulants, such as heparin. Cognitive impairment further contributes to bleeding risk through increased falls, cerebrovascular disease, medication nonadherence, and postoperative delirium. The Essential Frailty Toolset (EFT) includes serum albumin and haemoglobin, both of which influence bleeding risk. Hypalbuminaemia may amplify the anticoagulant effect, while anaemia reflects systemic inflammation or hyperdynamic states that predispose the patient to haemorrhage. Other key predictors include advanced age, female sex, CKD, and peripheral vascular disease [26]. Procedural risk is heightened with non-femoral access and larger sheath-to-artery ratios. Dual antiplatelet therapy is associated with a significantly higher bleeding rate compared to single antiplatelet therapy [27]. In terms of late bleeding, physiological contributors, such as von Willebrand factor (vWF) dysfunction, have emerged as key elements. Persistent shear stress from residual paravalvular leak (PVL) may impair vWF function and prevent restoration of primary haemostasis. An elevated closure time with adenosine diphosphate (CT-ADP > 180 s), a marker of vWF deficiency, has been shown to predict late major bleeding events and is mechanistically linked to residual PVL [26,29].

Prediction models such as PREDICT-TAVR incorporate haemoglobin, serum iron, creatinine clearance, femoral artery size, and antithrombotic therapy to estimate the 30-day bleeding risk. In validation studies, the highest-risk quartile had an incidence of bleeding of 8.5% compared to less than 1% in the lowest-risk group [26,31].

## 9. Biomarkers for Risk Stratification in TAVI

Blood biomarkers are increasingly crucial for risk-stratifying patients undergoing TAVI. Among these, natriuretic peptides, markers of myocardial injury, inflammatory mediators and indicators of haemostatic imbalance have been the most studied.

Elevated levels of B-type natriuretic peptide (BNP) before TAVI have consistently been associated with increased mortality and adverse outcomes. A meta-analysis including more than 8000 patients demonstrated that pre-procedural BNP strongly predicts both 30-day and midterm mortality [23]. Findings from the OCEAN-TAVI registry demonstrate that patients discharged with a BNP level above 202 pg/mL had significantly higher two-year rates of all-cause mortality and heart failure rehospitalisation [32]. Although BNP is clearly a marker of overall risk, its direct association with thromboembolic or bleeding complications is less well-established.

Troponin is another biomarker that has been evaluated. Troponin elevation after TAVI reflects procedural myocardial injury, and the VARC-3 consensus defines periprocedural myocardial injury as a substantial post-TAVI troponin rise within 48 h [33]. Baseline high-sensitivity troponin has also been shown to independently predict all-cause mortality after TAVI [34].

Inflammatory biomarkers, particularly high-sensitivity C-reactive protein (hs-CRP), also provide valuable prognostic information. Elevated hs-CRP at baseline is associated with significantly higher 30-day and one-year mortality, and with a higher incidence of complications, such as stroke, myocardial infarction and AKI, due to the risk of developing a systemic inflammatory response [35]. Risk appears to increase in a graded fashion; in one cohort, each 10 mg/L increase in hs-CRP was associated with a 14% higher risk of mortality. Other studies have demonstrated that patients in the highest tertile experience significantly greater one-year mortality [36]. The combination of hs-CRP with the logistic EuroSCORE has been shown to improve risk stratification, classifying patients into subgroups, with one-year mortality ranging from 6.6% in the low-risk group to 18.2% in the high-risk group [37]. These findings highlight the importance of considering systemic inflammation, especially in frail patients, when assessing morbidity and mortality after TAVI [36]. Specifically, regarding thrombosis, a smaller study has supported the prognostic value of hs-CRP for disabling stroke within one year [38].

Other novel biomarkers have been investigated. Growth Differentiation Factor-15 (GDF-15) has emerged as a strong predictor of both mortality and lack of left ventricular recovery, outperforming NT-proBNP for risk stratification. In contemporary cohorts, predefined cutoffs around 2000 pg/mL have been associated with significantly reduced survival [39,40]. Elevated galectin-3 levels (>17.8 ng/mL) have also been associated with poorer outcomes when used with NT-proBNP [41], and small studies have suggested that markers of oxidative stress, such as malondialdehyde, may be linked to adverse outcomes [42]. However, these data remain preliminary.

Haemostatic and platelet-related biomarkers are particularly relevant to bleeding and thrombotic risk. In severe AS, high shear stress across the stenotic valve results in loss of high-molecular-weight vWF multimers; activation of platelets with the release of P-selectin and platelet factor-4; and increased coagulation activity as evidenced by rises in D-dimer, thrombin–antithrombin complexes, and plasmin–antiplasmin complexes. This leads to a paradoxical state combining a prothrombotic milieu with a bleeding tendency. Following TAVI, persistent thrombocytopenia occurs in around a third of patients and is strongly associated with early complications and mortality at one year. This fall in platelet count is driven by platelet activation, and markers such as thrombin–antithrombin complexes, plasmin–α2–antiplasmin complexes, D-dimer, platelet factor-4 and P-selectin track this reduction. The inflammatory response further influences the thrombocytopenia, as increases in interleukin-6 and S100A8/A9 correlate closely with platelet drop. Regarding bleeding, platelet-specific markers, particularly a high mean platelet volume and low platelet distribution width, have been associated with an increased risk of significant bleeding. At the same time, recovery of high-molecular-weight vWF multimers after TAVI reflects restoration of usual shear forces and correlates with better outcomes. Taken together, these biomarker changes highlight the complex haemostatic disturbance after TAVI and may help to identify patients at higher risk of bleeding or thromboembolic complications [43].

Increasingly, studies are demonstrating that a multimarker approach provides greater predictive value. In a 362-patient study, patients with eight or nine abnormally elevated biomarkers before TAVI had markedly worse outcomes than those with fewer abnormalities. The biomarkers included in this panel covered multiple domains: markers of myocardial injury (creatine kinase MB, GDF-15, high-sensitivity cardiac troponin, NT-proBNP, human epididymis protein 4, and cancer antigen 125), markers of inflammation (interleukin-6, high-sensitivity CRP, ferritin, lactate dehydrogenase, soluble fms-like tyrosine kinase-1, procalcitonin, alkaline phosphatase and alanine aminotransferase), and markers of renal function (creatinine). While this study found no clear correlation between biomarker burden and post-procedural stroke or TIA, there was a trend towards more myocardial infarction in those with a higher number of elevated biomarkers [44].

Risk models incorporating biomarkers have been developed to identify patients at high risk of bleeding. The PREDICT-TAVR score combines laboratory variables (haemoglobin, iron, and creatinine clearance) with anatomical and treatment factors, such as femoral artery diameter and the use of dual antiplatelet or OAC. This score outperformed other tools, such as HAS-BLED and PARIS, in predicting 30-day bleeding complications, achieving an area under the curve (AUC) of 0.78 [31].

More recently, artificial intelligence has been applied to risk stratification after TAVI. A meta-analysis of 43 studies found that machine learning models that integrate biomarkers with clinical and imaging data outperform conventional approaches [45]. Specifically, for thromboembolic outcomes, this meta-analysis demonstrated a pooled AUC of 0.73 (95% CI: 0.59–0.88) for stroke and TIA. This was moderate compared to the performance for all-cause mortality, and only three studies contributed to the stroke models.

In summary, BNP and troponin remain validated biomarkers for mortality, whereas hs-CRP, GDF-15, galectin-3, and markers of platelet activation and coagulation are emerging as potential predictors of bleeding and thrombotic risk. Multimarker panels and machine learning models show promise in this field, especially when biomarkers are combined with clinical and imaging data. Evidence specifically linking biomarkers to post-TAVI stroke or major bleeding remains limited and requires further study.

## 10. Overview of Antithrombotic Therapy After TAVI

Randomised evidence supports an indication-driven strategy for antithrombotic therapy after TAVI, with treatment intensity determined primarily by the patient’s baseline need for OAC (Table 1). Two cohorts from the POPular TAVI programme provide the most precise clinical guidance. In patients without an indication for long-term OAC, aspirin monotherapy reduced 12-month bleeding compared with aspirin plus clopidogrel (15.1% vs. 26.6%), without a detectable increase in ischaemic events, supporting single antiplatelet therapy (SAPT) over routine dual antiplatelet therapy (DAPT) in most non-OAC TAVI patients [46]. In patients already receiving chronic anticoagulation, OAC alone reduced bleeding (21.7% vs. 34.6%) and preserved noninferiority for ischaemic outcomes compared with OAC plus clopidogrel, supporting OAC monotherapy when a formal anticoagulation indication exists [46]. Studies that tested routine anticoagulation in patients without an OAC indication produced cautionary or adverse signals. GALILEO, which randomised 1644 patients to rivaroxaban 10 mg daily plus short aspirin versus an antiplatelet strategy, was terminated early for safety after an excess of death and thromboembolic events and numerically increased major bleeding in the rivaroxaban arm [47]. ATLANTIS compared routine apixaban with the standard of care in an all-comer TAVI population. In the standard-of-care arm, patients with an indication for anticoagulation received a vitamin K antagonist (VKA), whereas those without an indication received antiplatelet therapy. The trial did not demonstrate superiority of apixaban for the composite clinical endpoint of 1 year. Of note, in patients without an indication for anticoagulation, there was an increased number of non-cardiovascular related deaths linked to the apixaban arm [48]. Together, these trials argue against routine direct oral anticoagulants (DOAC) in patients without established anticoagulation indications and underscore that agent and dosing choices materially affect outcomes.

When OAC is indicated, randomised data support OAC monotherapy over the routine addition of antiplatelet agents, but agent selection should account for bleeding risk. ENVISAGE-TAVI AF found edoxaban to be noninferior to VKA therapy for a composite clinical endpoint in predominantly AF patients after TAVI; however, edoxaban was associated with higher major bleeding (9.7 vs. 7.0 per 100 patient-years), notably gastrointestinal bleeding [50]. The ENVISAGE on-treatment sub-analysis reported a significant rate of gastrointestinal bleeding of 6.0% in the on-treatment cohort. They identified predictors concentrated among edoxaban recipients (lack of dose adjustment, recent percutaneous coronary intervention [PCI] ≤ 30 days, smoking, and low haemoglobin), implicating modifiable and agent-specific contributors to harm [49]. In particular, given the high prevalence of renal dysfunction in TAVI patients, advanced CKD confers increased bleeding risk and complicates antithrombotic management. Certain DOACs (e.g., edoxaban and rivaroxaban) are not recommended in severe renal impairment (eGFR < 15 mL/min), while others require dose adjustments [26,53]. These findings support OAC monotherapy for patients with AF while emphasising individualised agent selection and proactive bleeding mitigation (dose adjustment, gastrointestinal risk assessment, and consideration of proton-pump inhibitor prophylaxis where appropriate).

Mechanistic and imaging-focused trials demonstrate that anticoagulation can reduce CT-detectable leaflet abnormalities, but translating these findings into meaningful clinical benefits remains unproven. Roger et al. reported that a 30-day warfarin plus low-dose aspirin regimen reduced HALT and early valve dysfunction at 30 days compared with aspirin alone in a small, low-risk cohort, with no excess short-term bleeding [51]. Park et al. found a numerically lower rate of leaflet thrombosis with edoxaban compared with dual antiplatelet therapy at 6 months; however, no difference in new cerebral lesions, neurocognitive outcomes, or bleeding was detected, and the study was underpowered for clinical endpoints [24]. These observations indicate that while anticoagulation mitigates imaging surrogates of valve thrombosis, imaging improvements alone are insufficient justification for routine escalation of anticoagulation in the absence of demonstrable reductions in stroke, major adverse cardiac events (MACEs), or sustained valve dysfunction.

The POPular TAVI cohorts are adequately powered for bleeding endpoints and, therefore, highly applicable to routine practice; their consistent signal in both non-OAC and OAC strata provides strong evidence for a minimised, indication-based approach [46,52]. By contrast, GALILEO’s early termination reinforces a clear safety signal for the tested rivaroxaban regimen but limits mechanistic exploration [47]. ATLANTIS and ENVISAGE are large and informative but heterogeneous in design and background therapy, which complicates cross-trial comparisons and subgroup interpretation [48,50]. Imaging trials are informative for procedural mechanisms but are small, often lower risk or selected, and underpowered for clinical endpoints, limiting generalisability [24,51]. Available trials differ by anticoagulant agent (rivaroxaban, apixaban, edoxaban, VKA), dose (notably 10 mg of rivaroxaban in GALILEO), background antiplatelet strategy, and primary endpoint (clinical composites versus imaging surrogates). These differences imply that observed benefits or harms may be agent- or dose-specific rather than class effects; therefore, meta-analytic pooling must stratify by OAC indication, agent and dose to avoid misleading conclusions.

Furthermore, several trials report that most bleeding occurs early (periprocedural to first month), emphasising that early post-TAVI management and transient exposures drive much of the net harm and that short-course strategies may have different risk–benefit profiles than chronic regimens [46,52]. Importantly, early post-TAVI bleeding (particularly within the first 30 days) has consistently been shown to be one of the strongest predictors of early mortality, underscoring the importance of minimising unnecessary antithrombotic intensity during this vulnerable period [28]. Additionally, the excess mortality and thromboembolic signal in GALILEO, and the excess gastrointestinal bleeding with edoxaban in ENVISAGE, represent clinically meaningful, reproducible concerns that should influence prescribing and trial design. These signals caution against mechanistic extrapolation from imaging benefits to overall net clinical benefits and argue for agent-level safety assessments rather than class-wide assumptions [47,49,50].

Observed treatment effects are consistent for key pragmatic comparisons: SAPT versus DAPT in non-OAC patients (reduced bleeding with SAPT) and OAC alone versus OAC plus antiplatelet in OAC patients (reduced bleeding with OAC monotherapy). For most contemporary TAVI patients, a risk-stratified default strategy is supported: SAPT (aspirin) for patients without OAC indication [46,47]; OAC monotherapy for patients with AF or other validated anticoagulation indications [48]; avoidance of routine DOAC strategies in unselected non-OAC patients outside clinical trials [24]; and individualised agent choice with attention to bleeding risk where OAC is required [50]. Imaging surveillance for subclinical leaflet thrombosis should remain primarily a research tool until trials demonstrate that imaging-driven therapy improves hard clinical outcomes.

Current ESC and AHA/ACC recommendations are based on trial evidence (Table 2). Both societies now advocate SAPT for patients without an indication for long-term anticoagulation and recommend OAC monotherapy for patients with AF, reserving combined therapy for select cases, such as those with recent PCI [54,55]. Importantly, neither guideline supports routine anticoagulation solely for the prevention of leaflet thrombosis, reflecting the discrepancy between radiological improvements and clinical outcomes in imaging trials [54,55]. However, guidance remains intentionally broad due to heterogeneity in trial designs, differences in DOAC safety profiles, and the underrepresentation of high-risk subgroups. As a result, clinical decision-making still requires individualised assessment, particularly in patients with frailty, high bleeding risk, or procedural complexity [54,55,56]. Notably, anaemia and frailty-associated markers—such as a low body mass index, comorbidities, and advanced age—are incorporated into contemporary consensus definitions (e.g., VARC-HBR) aimed at identifying TAVI patients at high bleeding risk. This further supports minimizing anticoagulation intensity in vulnerable patients [57].

Several ongoing trials are directly addressing the key uncertainties highlighted in this review. ACLO-TAVR (NCT05493657) is enrolling TAVI patients without an indication for OAC and compares aspirin with clopidogrel monotherapy after an initial 4 weeks of dual antiplatelet therapy, with the primary endpoint being CT-identified leaflet thrombosis at 3 months. Similarly, ACASA-TAVI (NCT05035277) is evaluating a DOAC-based strategy versus aspirin in patients aged 65–80 years without an indication for OAC, assessing the incidence of HALT on CT as well as a composite of bleeding, thromboembolic events, and all-cause mortality at 12 months [58,59]. POPular ATLANTIS (NCT06168370) is investigating a CT-guided, personalised antithrombotic strategy compared with lifelong single antiplatelet therapy in patients without an indication for OAC, with treatment escalation reserved for those with radiological evidence of SLT [60]. In patients requiring long-term OAC, AVATAR (NCT02735902) is comparing OAC alone versus OAC plus aspirin, with a primary composite endpoint of bleeding and thromboembolic complications at 12 months. POPular PAUSE TAVI (NCT04437303) is evaluating whether continuation versus temporary interruption of chronic OAC around the time of TAVI affects a composite of cardiovascular death, stroke, myocardial infarction, major vascular complications, and major bleeding at 30 days [61,62]. Finally, the Non-antithrombotic Therapy After Transcatheter Aortic Valve Implantation (NAPT) trial (NCT06007222) is assessing the safety of complete antithrombotic withdrawal compared with single antiplatelet therapy in patients at very high bleeding risk [63]. Together, these studies will help clarify optimal long-term treatment for patients with and without indication for anticoagulation and determine whether an imaging-guided approach can improve clinical outcomes after TAVI.

## 11. Conclusions

TAVI has transformed the management of severe AS and is now performed across a broad patient spectrum, many of whom are elderly, frail, and highly comorbid. As its use has expanded, defining the optimal antithrombotic strategy has become increasingly complex. The evidence highlights the persistent challenge of balancing thromboembolic prevention against bleeding risk in a population predisposed to both.

Most thrombotic complications after TAVI are driven by peri-procedural factors and are largely unaffected by long-term antithrombotic therapy. In contrast, AF, particularly when persistent or occurring after discharge, remains a key determinant of adverse outcomes and warrants appropriate oral anticoagulation. SLT is frequently detected on imaging, but its clinical relevance is uncertain, and anticoagulation based solely on radiological findings has not been shown to improve patient-centred outcomes. Bleeding is a significant cause of early morbidity and mortality following TAVI, predominantly occurring within the first 30 days and strongly influenced by frailty and procedural factors. This vulnerability highlights the potential harm of aggressive antithrombotic strategies, particularly in the immediate post-procedural period.

Across randomised trials, treatment minimisation provides the most favourable balance of safety and efficacy. SAPT is preferred over DAPT in patients without an indication for anticoagulation, and OAC alone is superior to combination therapy in those with AF. Escalation strategies using DOAC have not demonstrated clinical benefits and are associated with excess bleeding, despite reducing imaging-detected leaflet abnormalities.

Overall, current data support antithrombotic strategies that prioritise bleeding avoidance while maintaining protection against established thrombotic risks. Routine treatment escalation, prolonged combination therapy, or anticoagulation guided solely by subclinical imaging findings cannot be justified. Future progress will depend on improved individual risk stratification, including the potential role of biomarkers and imaging-guided approaches.

## Figures and Tables

**Table 1:** Randomised clinical trials and key outcomes of antithrombotic strategies after TAVI. VKA: vitamin K antagonist, MACEs: major adverse cardiovascular events, ITT: intention-to-treat, AF: atrial fibrillation, TAVI: transcatheter aortic valve implantation, OAC: oral anticoagulation, DOAC: direct oral anticoagulant, CV: cardiovascular, MI: myocardial infarction, HR: hazard ratio, MGIB: major gastrointestinal bleeding.

Trial (Year)	Population (Number)	Follow-Up	Intervention (Agent/Regimen)	Comparator (Agent and Regimen)	Risk of Death	Bleeding	MACE/Thromboembolic Events
ENVISAGE on-treatment sub-analysis (Dangas 2025) [49]	1377 (on-treatment analysis)	Not stated	Edoxaban (on-treatment analysis)	VKA (on-treatment comparator)	Not primary focus	Major gastrointestinal bleeding occurred in 6.0% of patients (83/1377); 67.5% of MGIB events occurred on edoxaban	Not primary focus
ATLANTIS (2022) [48]	1500 (749 apixaban; 751 standard care)	1 year	Apixaban 5 mg twice daily (dose-reduced where indicated)	Standard of care: VKA if indicated; antiplatelet therapy if not	No overall difference between apixaban and standard care	Primary safety endpoint was similar between groups	No superiority for composite outcomes; overall, MACE was comparable
Park et al. (2022) [24]	229 (final ITT population)	Primary imaging endpoint at 6 months	Edoxaban	Dual antiplatelet therapy (aspirin + clopidogrel)	No difference reported	No difference in any or major bleeding	No difference in new cerebral lesions or neurocognitive outcomes
ENVISAGE-TAVI AF (Van Mieghem 2021) [50]	1426 (713 edoxaban; 713 VKA)	Follow-up not explicitly stated	Edoxaban (label dosing)	Vitamin K antagonist (warfarin)	No clear excess mortality; rates of death/stroke were similar	Higher major bleeding with edoxaban: 9.7 vs. 7.0 per 100 patient-yrs (HR 1.40)	Composite primary efficacy was similar; edoxaban was noninferior (HR: 1.05)
Rogers et al. (2021) [51]	94 randomized (50 aspirin; 44 warfarin + aspirin) plus 30 registry patients	30 days	Warfarin + low-dose aspirin (30 days)	Low-dose aspirin alone	No excess short-term mortality reported	No excess bleeding at 30 days with short warfarin regimen	Primary imaging composite was lower with a short warfarin regimen
POPular TAVI Cohort A (Brouwer 2020) [46]	665 (331 aspirin; 334 aspirin + clopidogrel)	12 months	Aspirin alone	Aspirin + clopidogrel (3 months)	No increase reported	Lower with aspirin alone: 15.1% vs. 26.6%	Composites (CV death, non-procedure bleeding, stroke, and MI) were lower or noninferior with aspirin alone
POPular TAVI Cohort B (Nijenhuis 2020) [52]	313 (157 OAC; 156 OAC + clopidogrel)	12 months	OAC alone (warfarin or DOAC as clinically indicated)	OAC + clopidogrel (3 months)	No increase reported	Lower with OAC alone: 21.7% vs. 34.6%; most bleeding was early and minor	Secondary composites (CV death, non-procedure bleeding, stroke, and MI) were lower/noninferior with OAC alone
GALILEO (Dangas 2020) [47]	1644	Median 17 months	Rivaroxaban 10 mg daily + aspirin	Antiplatelet strategy: aspirin ± clopidogrel (3 months)	Higher death in rivaroxaban arm (64 vs. 38 deaths reported)	Numerically higher major/disabling bleeding with rivaroxaban (HR: 1.50; *p* = 0.08)	Higher composite of death or thromboembolic events with rivaroxaban (HR: 1.35; *p* = 0.04)

**Table 2:** Comparison of ESC and AHA/ACC guideline recommendations for post-TAVI antithrombotic therapy. TAVI: transcatheter aortic valve implantation, ESC: European Society of Cardiology, AHA: American Heart Association, ACC: American College of Cardiology, OAC: oral anticoagulation, SAPT: single antiplatelet therapy, DAPT: dual antiplatelet therapy, AF: atrial fibrillation, VKA: vitamin K antagonist, DOAC: direct oral anticoagulant, PCI: percutaneous coronary intervention, P2Y12: adenosine diphosphate P2Y12 receptor, NVAF: non-valvular atrial fibrillation.

Post-TAVI Clinical Scenario	ESC [54]	AHA/ACC [55,56]
No formal OAC indication	SAPT, usually aspirin, is preferred over routine DAPT; routine anticoagulation not recommended.	SAPT is preferred over DAPT for most patients. Routine anticoagulation for all is not recommended.
Established OAC indication (e.g., AF)	Continue OAC alone (VKA or DOAC) rather than routinely adding antiplatelet therapy. If recent PCI, individualise and minimise overlap duration.	OAC monotherapy is recommended in most AF patients after TAVI. Add antiplatelet only for specific, time-limited indications (e.g., recent PCI).
Recent PCI/recent coronary stent	This is a multidisciplinary, individualised plan. If combined therapy is unavoidable, keep the duration as short as possible and prioritise bleeding mitigation.	Balance stent thrombosis risk vs. bleeding: use shortest effective combined therapy and prefer clopidogrel when a P2Y12 inhibitor is required.
Choice of anticoagulant when OAC is required (VKA vs. DOAC)	DOACs are acceptable for non-valvular AF after TAVI, but consider agent-specific bleeding profiles and ensure appropriate dose adjustment.	DOACs are generally preferred for NVAF, but clinicians should consider DOAC-specific bleeding risks and individual patient factors.

## Data Availability

Not applicable.
